# Prognostic Stratification and Subtyping of Glioblastoma Using Transient Receptor Potential Channels

**DOI:** 10.1155/humu/7039302

**Published:** 2026-04-19

**Authors:** Runfeng Sun, Long Zhu, Zhichao Lu, Ziheng Wang, Suyin Feng, Jingwei Zhao

**Affiliations:** ^1^ Cardio-Cerebral Vascular Disease Prevention and Treatment Innovation Center, Donghai County People′s Hospital, Lianyungang, Jiangsu, China; ^2^ Department of Neurosurgery, Donghai County People′s Hospital, Lianyungang, Jiangsu, China; ^3^ Department of Neurosurgery, Research Center of Clinical Medicine, Affiliated Hospital of Nantong University, Medical School of Nantong University, Nantong, Jiangsu, China, ntu.edu.cn; ^4^ MOE Frontier Science Centre for Precision Oncology, University of Macau, Macau, China, umac.mo; ^5^ Department of General Surgery, Xinhua Hospital, Shanghai Jiao Tong University School of Medicine, Shanghai, China, shsmu.edu.cn

**Keywords:** glioblastoma, IFNGR2, immune infiltration, immunotherapy response, multiomics integration, prognostic model, TRP channels

## Abstract

**Background:**

Transient receptor potential (TRP) channels regulate Ca^2+^ homeostasis and tumor malignant phenotypes, whereas their prognostic relevance and therapeutic implications in glioblastoma (GBM) remain poorly characterized.

**Methods:**

We curated a comprehensive compendium of 522 TRP‐related genes from MSigDB, KEGG, and GeneCards. Differential expression analysis across clinical variables in The Cancer Genome Atlas Glioblastoma cohort (TCGA‐GBM; *n* = 529) identified 193 TRP‐associated genes significantly linked to patient outcomes. Using univariate Cox regression followed by LASSO‐Cox regularization, we developed a seven‐gene transient receptor potential‐related prognostic risk score (TRPRS). The model was rigorously validated in three independent external cohorts. Integrated multiomics analyses encompassed genomic alterations, tumor immune microenvironment profiling, and drug sensitivity prediction. Functional validation focused on the top‐ranked gene, IFNGR2, using in vitro glioma models.

**Results:**

TRPRS robustly stratified GBM patients into high‐ and low‐risk groups with significantly distinct overall survival across all four datasets (AUC = 0.72–0.81). Genomically, high‐TRPRS tumors were enriched for PTEN loss and 9q21.3 amplification, whereas low‐TRPRS tumors frequently harbored TP53 mutations and 1q21.3 deletions. High TRPRS was associated with diminished cytotoxic T‐cell infiltration and predicted resistance to multiple therapeutics—including cisplatin, carmustine, gefitinib, buparlisib, and afatinib. Notably, TIDE analysis revealed significantly reduced likelihood of response to immune checkpoint blockade in high‐TRPRS GBM, a pattern consistently observed in immunotherapy‐treated cohorts of melanoma, renal cell carcinoma, and bladder cancer. Functional assays demonstrated that IFNGR2 knockdown suppressed glioma cell proliferation and attenuated NF‐*κ*B signaling, underscoring its role as a key driver within the TRP network.

**Discussion:**

TRPRS provides a robust, biologically grounded tool for simultaneous prognostication and therapy guidance in GBM, highlighting TRP signaling as a therapeutic vulnerability.

## 1. Introduction

GBM is the most common and lethal primary intracranial malignancy in adults [1, 2]. Despite maximal safe surgical resection followed by concurrent radiotherapy with temozolomide and adjuvant chemotherapy, median overall survival remains dismally short—typically less than 15 months [3–6]. This grim prognosis stems from profound intratumoral heterogeneity, diffuse infiltration along white matter tracts, and near‐universal development of therapy resistance, which collectively preclude complete tumor resection and drive inevitable local recurrence [7, 8]. These challenges highlight an urgent need for robust molecular stratification tools capable of refining prognostic prediction and guiding personalized therapeutic strategies.

Recent transcriptomic and functional studies have implicated the TRP channel family, a group of nonselective cation channels, in GBM pathobiology. Across multiple independent cohorts, dysregulated expression of specific TRP isoforms correlates with aggressive tumor phenotypes, and experimental perturbation in vitro demonstrates that TRP‐mediated Ca^2+^ influx modulates key oncogenic processes, including glioma cell migration, angiogenic sprouting, and immune evasion [9, 10]. Nevertheless, the prognostic utility of a comprehensive TRP‐centric gene signature has not been systematically evaluated, and the relative contributions of individual TRP family members to GBM progression remain poorly defined.

The mammalian TRP superfamily comprises 28 genes, organized into seven subfamilies (TRPA, TRPC, TRPM, TRPML, TRPN, TRPP, and TRPV) that transduce diverse environmental cues into cellular responses [11, 12]. Beyond classical sensory functions, TRP channels regulate proliferation, apoptosis, and metabolic reprogramming in both normal and malignant tissues [13]. In GBM, altered expression of TRPC1, TRPM2, TRPM7, and TRPV2 has been linked to tumor grade and patient outcome. [14–16]. However, a unified, clinically applicable prognostic model based on the broader TRP signaling network has yet to be established.

Among TRP channels, transient receptor potential vanilloid 1 (TRPV1), the capsaicin receptor, has garnered particular interest. Although high‐dose capsaicin initially induces acute pain, it subsequently leads to prolonged desensitization and analgesia, a phenomenon leveraged in cancer pain management: resiniferatoxin, a potent TRPV1 agonist, achieved > 30% pain reduction in Phase I/II clinical trials involving patients with advanced malignancies [17–21]. Emerging evidence now positions TRPV1 at the interface of nociception and oncogenesis. TRPV1 activation modulates intracellular Ca^2+^ dynamics, thereby influencing glioma cell invasion and stem‐like properties [12, 21]. Despite these insights, the prognostic relevance of TRPV1‐centered transcriptional signatures in GBM remains unexplored.

Building upon these observations, we hypothesized that an integrative TRP‐related gene signature could significantly improve risk stratification in GBM. To test this, we curated a compendium of 522 TRP‐centric genes from authoritative public repositories, constructed a 22‐gene TRP‐related prognostic risk score (TRPRS) through machine learning approaches, and validated its predictive accuracy across multiple independent datasets. We further investigated the associations between TRPRS and key molecular features (e.g., MGMT methylation and IDH1 mutation status), tumor immune microenvironment composition, and predicted responses to chemotherapy, targeted agents, and immunotherapy. Our results establish TRPRS as a biologically grounded, clinically actionable tool for precision oncology in GBM and provide a rationale for targeting TRP‐mediated signaling pathways in future therapeutic trials.

## 2. Materials and Methods

### 2.1. Data Acquisition and Cleaning

TCGA‐GBM expression, survival, and phenotype files were grabbed from UCSC Xena (log2‐affy‐RMA processed). CGGA‐mRNAseq‐693/325 and GEO sets GSE13041/GSE16011 served as validation pools. TRP gene lists were harvested from MsigDB (REACTOME_TRP_CHANNELS, *n* = 28), KEGG pathway hsa04750 (*n* = 44) and GeneCards keyword “transient receptor potential” (score > 20, *n* = 486); duplicates were removed to yield 522 factors. The data information was shown in Table 1.

**Table 1:** Sample information for training and validation sets.

	Data sources	Dataset number	Normal control group	Tumor tissue group
Training sets	TCGA	GBM	10	529
Validation sets	TCGA	mRNAseq_693	/	693
		mRNAseq_325	/	325
	GEO	GSE4412	/	85
		GSE13041	/	191

### 2.2. Tumor‐Specific TRP Expression Profiling

Limma (|*l* *o* *g* *F* *C*| > 0.3, *a* *d* *j*.*P* < 0.05) pinpointed 6317 DEGs (2122 up and 4195 down) between tumor and adjacent normal tissue, 193 of which were TRP‐related. Volcano plots, heatmaps, and a STRING PPI network (193 nodes, 2713 edges, PPI enrichment *p* < 1.0*e* − 16) visualized the dysregulation.

### 2.3. Unsupervised TRP Subtyping

ConsensusClusterPlus (maxK = 4, km, Pearson) identified k = 2 as the optimal partition; samples were assigned to Cluster 1 (*n* = 254) or Cluster 2 (*n* = 275) for subsequent analyses.

### 2.4. Construction of TRPRS

Prognosis‐linked TRP genes were first filtered by univariate Cox (*p* < 0.01); LASSO‐Cox then shrank the list to seven features and their coefficients. Patients were split by the median into high‐ and low‐TRPRS strata. Performance was tested in training and external sets.

TRPRS=Σi=1nCoefi∗Expri

where *Expr* represents the expression value of the feature gene in the model, and *Coef* represents the regression coefficient. Patients were stratified into high‐ and low‐risk groups based on the median risk score of all samples, and the prognostic efficacy of TRPRS was evaluated in both the training set and an external independent validation set.

### 2.5. TRPRS Versus Clinical Characteristics

ggpubr mapped TRPRS distributions across age, sex, IDH, 1p/19q, MGMT methylation, and molecular subtypes; immune infiltration and checkpoint expression were compared between risk groups.

### 2.6. Drug‐Sensitivity and Immunotherapy Prediction

pRRophetic estimated IC50 values for chemotherapeutics; TIDE web‐server computed immune‐escape likelihood. Analyses were repeated in melanoma (GSE91061), renal cell carcinoma, and bladder cancer (IMvigor210) immunotherapy cohorts.

### 2.7. Multiomics Integration

maftools compared mutation landscapes; GISTIC 2.0 online tool delineated copy number alterations between high‐ and low‐TRPRS tumors.

### 2.8. Cell Culture and Transient Transfection

U87‐MG cells (RRID:CVCL_0022), U251 (RRID:CVCL_0021), and normal HA glial cells were purchased from Wuhan Procell Life Science and Technology Co. Ltd.

U87‐MG population represents the commercially available U‐87 MG strain, which has been genetically distinct from the original 1966 isolate (PMID: 27182558). Cells were used within 10 passages after thawing and tested negative for mycoplasma contamination by PCR every 8 weeks. STR analysis of Wuhan Procell Life Science and Technology Co. Ltd. confirmed 100% consistency with the ExPASy U‐87 MG reference. All cell lines were maintained in DMEM supplemented with 10% fetal bovine serum and 1% penicillin–streptomycin at 37°C, 5% CO_2_. si‐IFNGR2 or negative control were transfected with Lipofectamine 2000.

### 2.9. qRT‐PCR and Western Blot

Total RNA was extracted with TRIzol, reverse‐transcribed (2 *μ*g), and amplified on a Roche LightCycler 480. Relative expression was calculated by 2^−*Δ* *Δ* *C* *t*^ with GAPDH as reference. RIPA lysates were resolved on 8% SDS‐PAGE, transferred to 0.45‐*μ*m PVDF, blocked (Beyotime quick block), probed overnight at 4°C with primary antibodies (Table), and incubated with HRP‐conjugated secondary antibodies for 1 h at RT. Bands were visualized with ECL (Beyotime) on a Tanon 4600 system. Primer pairs and antibody information were listed in the Supporting Information.

### 2.10. CCK‐8 and EdU Assays

Cells were seeded at 1 × 10^3^ per well in 96‐well plates; 10‐*μ*L CCK‐8 reagent was added at indicated time‐points and OD450 measured after 2 h incubation (BioTek reader). EdU (10 *μ*M) was pulsed for 2 h, cells were fixed, permeabilised, and stained according to the Beyotime kit protocol; EdU‐positive nuclei were counted under fluorescence microscopy.

### 2.11. Statistical Analysis

All statistical computations were performed with R v4.2.3 (packages survminer, survival, glmnet, pRRophetic, ConsensusClusterPlus, maftools, and GSVA), SPSS 27.0 (IBM Corp.), and GraphPad Prism 9.5. Categorical variables are reported as *n* (%) and continuous variables as mean ± SD or median (IQR). Differential expression was tested with limma voom (FDR‐adjusted *p* < 0.05). Survival curves were estimated by Kaplan–Meier method and compared with log‐rank test. Cox proportional‐hazards assumptions were verified by Schoenfeld residuals (global *p* > 0.05). Univariable and multivariable Cox models report hazard ratios (HRs) with 95% confidence intervals (CIs). Harrell′s C‐index and time‐dependent ROC (survivalROC, 1–5 years) were used to quantify discriminative accuracy; AUC comparison between nested models employed DeLong′s test. Consensus clustering stability was assessed by cophenetic correlation and average silhouette width. Drug‐sensitivity predictions were summarized as mean ± SEM; IC50 differences were evaluated by two‐sided Wilcoxon rank‐sum test with Benjamini–Hochberg correction. Immune‐infiltration and checkpoint correlations were analyzed by Spearman *ρ* with FDR < 0.05. All tests are two‐tailed; *p* < 0.05 was considered statistically significant unless otherwise specified. Multiple‐comparison corrections were applied where indicated.

## 3. Results

### 3.1. Data Acquisition and Preprocessing of GBM and TRP‐Related Genes

The REACTOME_TRP_CHANNELS gene set (*n* = 28) was downloaded from MSigDB; 44 genes were extracted from the KEGG “inflammatory mediator regulation of TRP channels” pathway (hsa04750). A keyword search of “transient receptor potential” in GeneCards returned 11,471 entries; filtering for relevance score > 20 retained 486 genes. Union of the three sources yielded 522 nonredundant TRP‐related factors.

### 3.2. Dysregulation Landscape of TRP Genes in GBM

Volcano and heatmap visualization highlighted 193 significantly dysregulated TRP genes in tumor versus nontumor tissue (Figure 1a,b). Stratification by clinical attributes revealed that these genes were also differentially expressed according to 1p/19q codeletion, MGMT promoter methylation, classical/mesenchymal/proneural subtypes, and IDH1 mutation status (Figures 1c, 1d, 1e, and 1f). A PPI network constructed with the 193 differential genes (STRING default settings) showed dense connectivity, with > 90% of nodes possessing ≥ 1 interacting partner (Figure 1g).

Figure 1TRP‐related genes are aberrantly expressed in glioblastomas and are associated with clinical features. (a) Volcano plot of differentially expressed genes. (b) Heatmap of differentially expressed genes. (c) Heatmap of expression levels of 193 differentially expressed TRP factors in relation to 1p_19q codeletion. (d) Heatmap of expression levels of 193 differentially expressed TRP factors in relation to MGMT methylation status. (e) Heatmap of expression levels of 193 differentially expressed TRP factors in relation to molecular subtype (original subtype). (f) Heatmap of expression levels of 193 differentially expressed TRP factors in relation to IDH1 mutation status. (g) Protein–protein interaction (PPI) network diagram of 193 TRP genes (green represents genes with logFC < 0 and red represents genes with logFC > 0).

**Figure:**
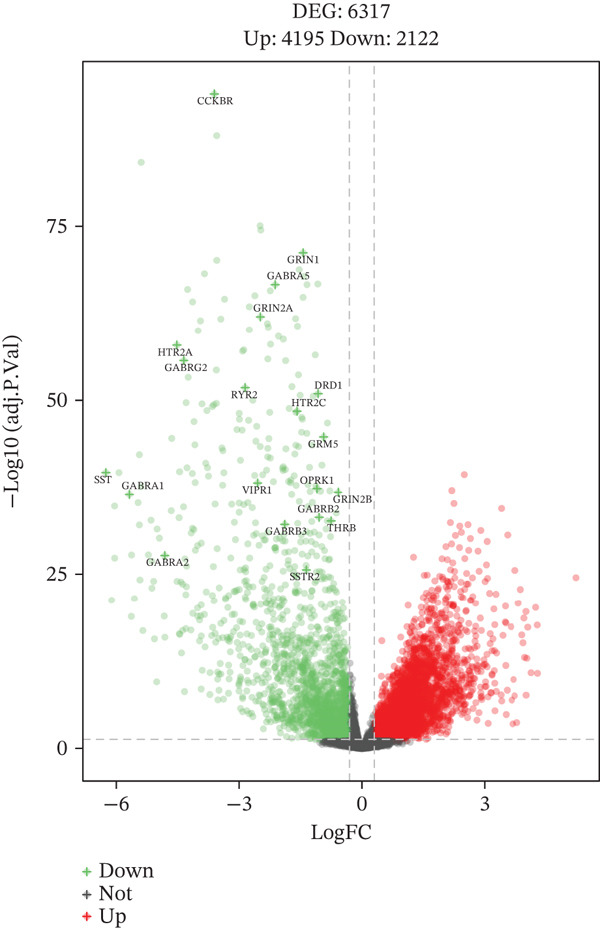
(a)

**Figure:**
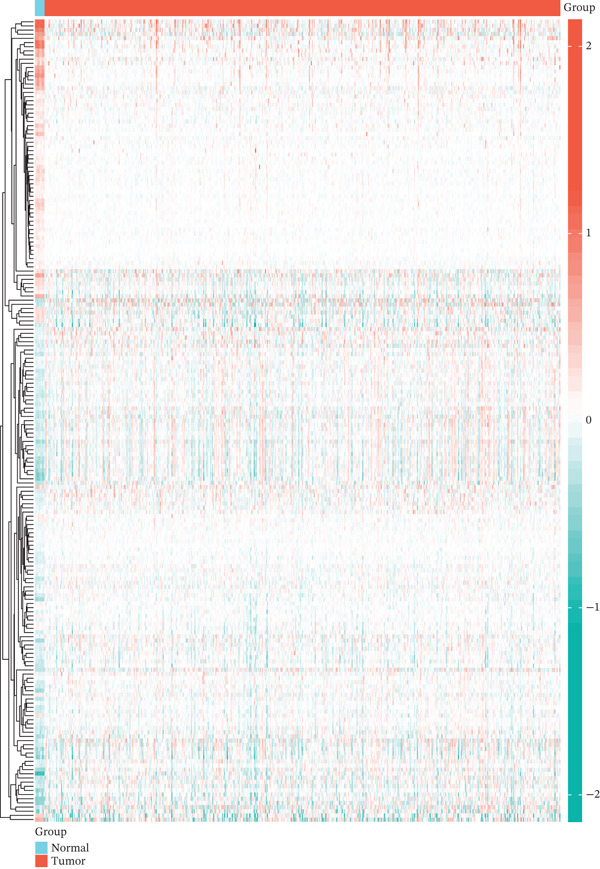
(b)

**Figure:**
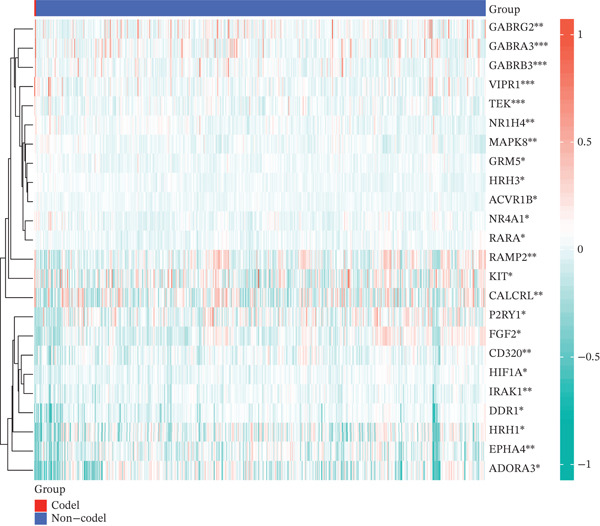
(c)

**Figure:**
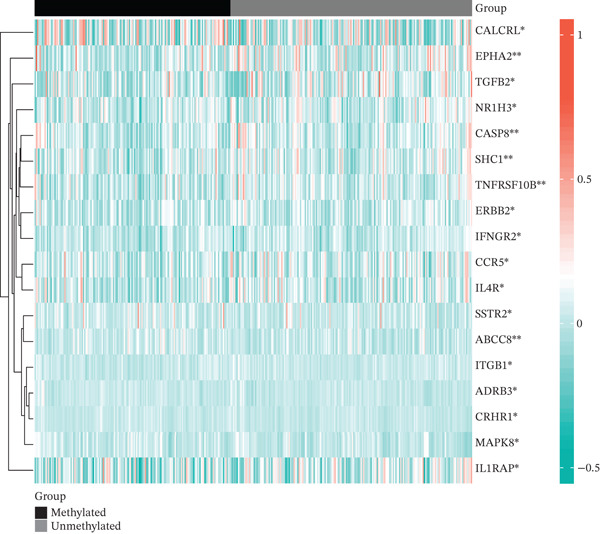
(d)

**Figure:**
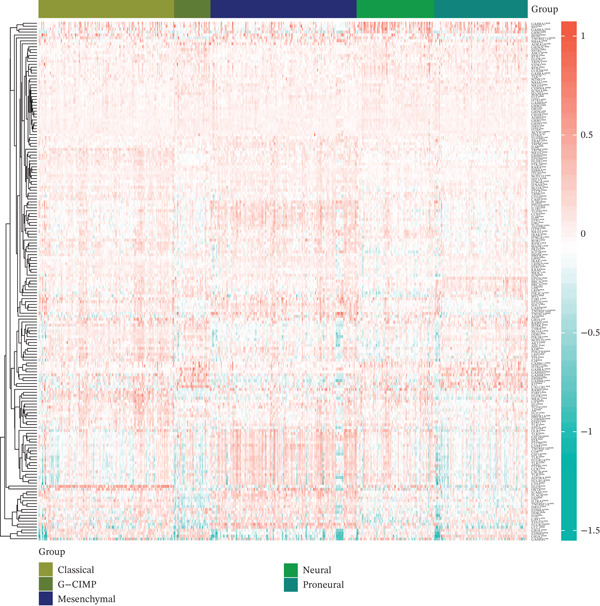
(e)

**Figure:**
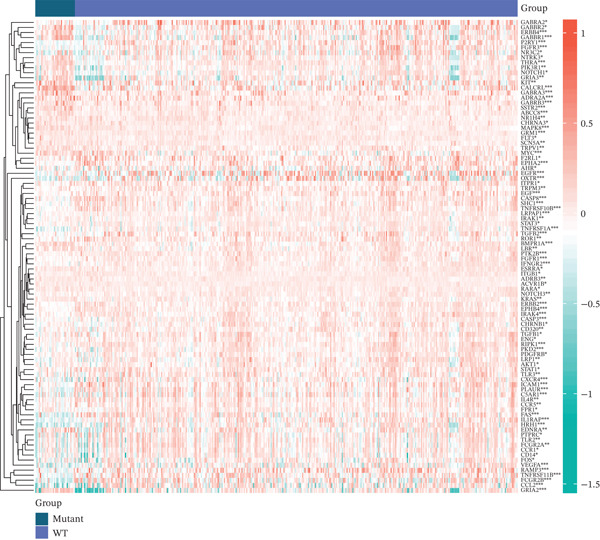
(f)

**Figure:**
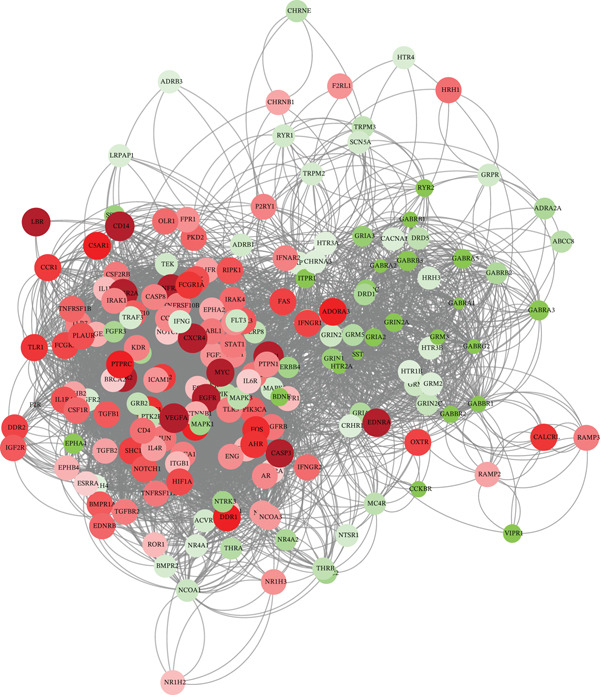
(g)

### 3.3. TRP‐Based Molecular Subtypes Correlate With Clinical Features and Immune Infiltration

Consensus clustering (k = 2) optimally partitioned 529 TCGA‐GBM samples into Cluster 1 (*n* = 254) and Cluster 2 (*n* = 275) (Figure 2a,b). Survival differed significantly between clusters (log‐rank *p* = 0.0079; Figure 2c). Clinical–pathologic comparison demonstrated associations of TRP subtypes with age, sex, IDH status, 1p/19q codeletion, MGMT methylation, and classical/mesenchymal/proneural classification (*p* < 0.05; Figure 2d). Immune‐cell quantification (CIBERSORT) revealed distinct infiltration patterns for CD8^+^ T cells, naïve CD4^+^ T cells, activated dendritic cells, eosinophils, and neutrophils (Figure 2e).

Figure 2Association of TRP‐related gene molecular subtypes with clinical features and immune infiltration. (a) Heatmap of the matrix for k = 2, with both rows and columns representing samples. (b) Cumulative distribution function (CDF) plot showing the CDF for different values of k. (c) Prognostic survival analysis between subgroups. (d) Heatmap of correlations between TRP subtypes and clinical features. (e) Analysis of immune infiltration differences between subtypes ( ^∗^ *p* < 0.05;  ^∗∗^ *p* < 0.01;  ^∗∗∗^ *p* < 0.001; and  ^∗∗∗∗^ *p* < 0.0001; ns *p* > 0.05).

**Figure:**
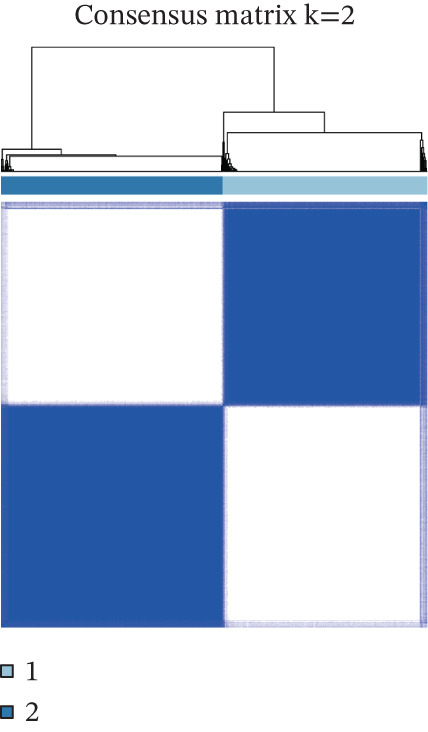
(a)

**Figure:**
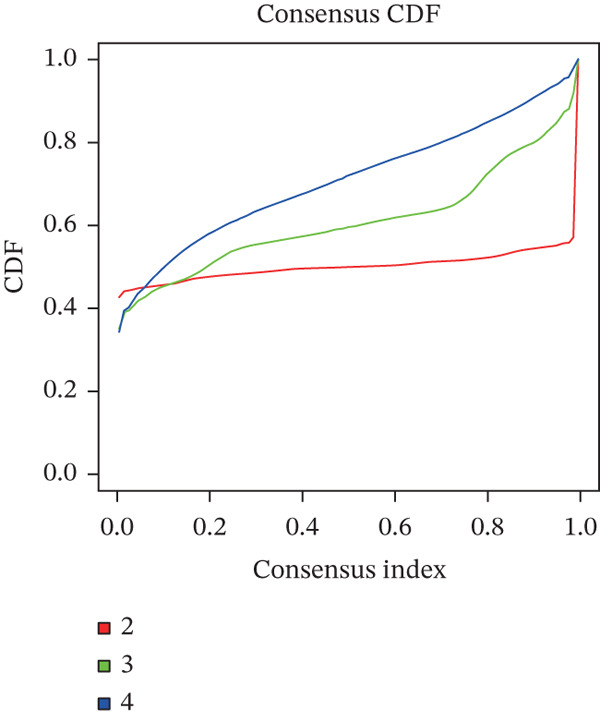
(b)

**Figure:**
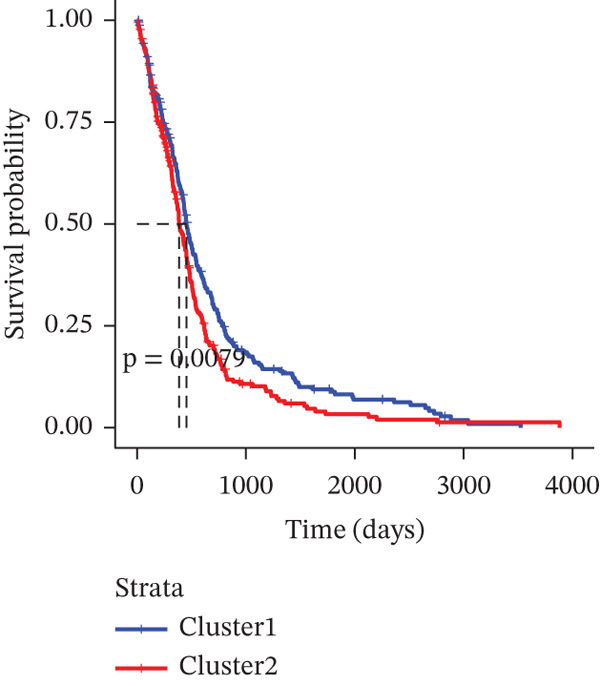
(c)

**Figure:**
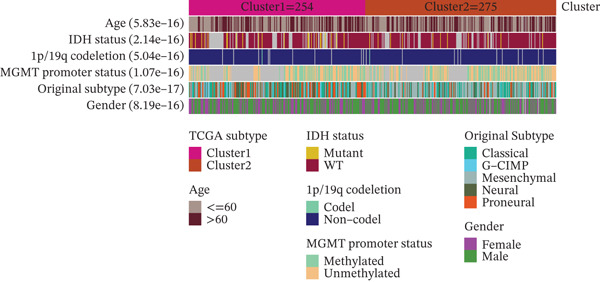
(d)

**Figure:**
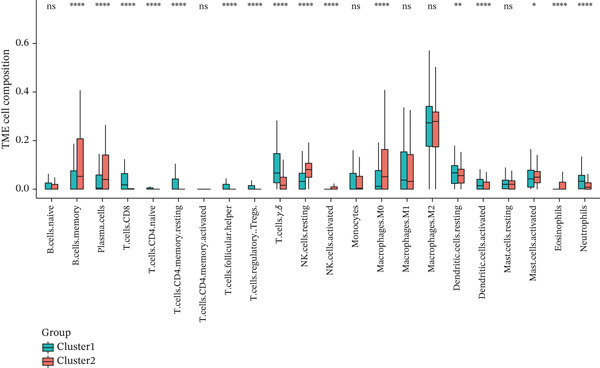
(e)

### 3.4. Development and Validation of the TRPRS

Comparing the two clusters, 60 TRP genes met |log2*F* *C*| > 0.5 and FDR < 0.05. Univariate Cox regression (*p* < 0.01) filtered 22 prognostic candidates; the top four are displayed in Figure 3a. LASSO‐Cox regularization retained seven genes and their coefficients (Figure 3b,c, Table 2), with which TRPRS was calculated for each patient. In the training set, risk stratification by median TRPRS produced markedly divergent survival curves (*p* < 0.0001) with ROC − AUC = 0.75 (Figure 4a). Independent validation cohorts yielded comparable discrimination: CGGA‐mRNAseq‐693: AUC = 0.717, *p* < 0.001 (Figure 4b); CGGA‐mRNAseq‐325: AUC = 0.814, *p* < 0.0001 (Figure 4c); GSE13041: AUC = 0.562, *p* = 0.29 (Figure 4d); GSE4412: AUC = 0.738, *p* = 0.022 (Figure 4e). Univariate and multivariate Cox analyses confirmed TRPRS as an independent predictor of overall survival after adjustment for age and sex in TCGA‐GBM, CGGA‐693, and CGGA‐325 (HR> 1, *p* ≤ 2.7 × 10 − 6; Figures 5a, 5b, and 5c); no significant independence was observed in GSE13041 or GSE4412 (Figure 5d,e).

Figure 3Construction of TRP‐related prognostic stratification scoring system. (a) Survival prognostic curves for the top four most significant prognostic genes. (b) The lowest point on the curve indicates the optimal lambda. (c) Bar plot of the seven feature genes and their regression coefficients.

**Figure:**
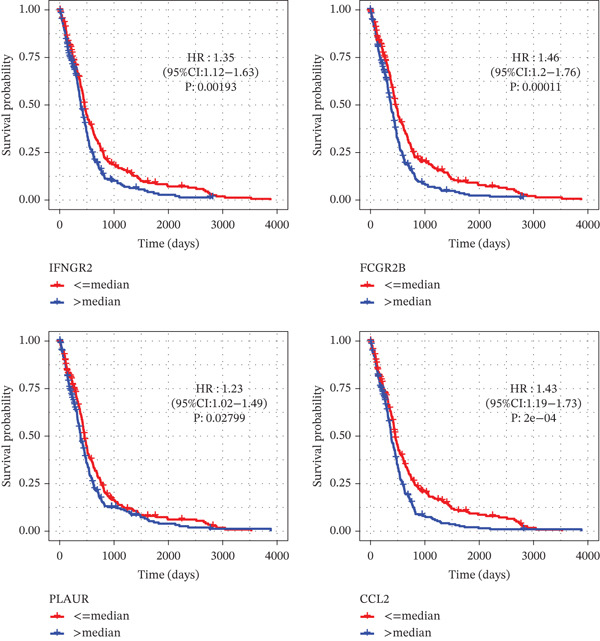
(a)

**Figure:**
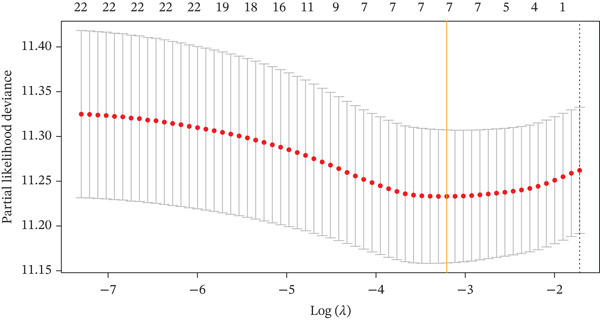
(b)

**Figure:**
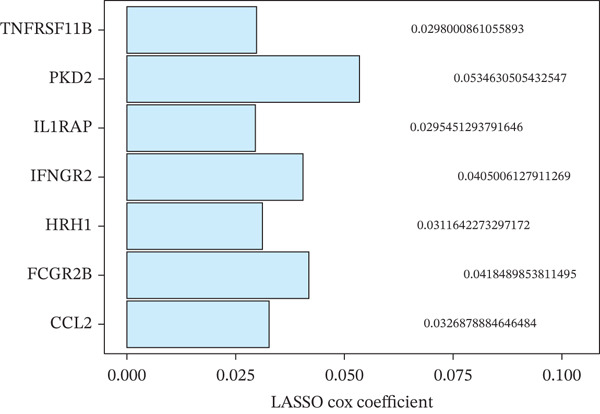
(c)

**Table 2:** Seven characteristic genes and regression coefficients.

Gene	Active coefficients
FCGR2B	0.041849
CCL2	0.032688
IFNGR2	0.040501
HRH1	0.031164
PKD2	0.053463
TNFRSF11B	0.029801
IL1RAP	0.029545

Figure 4Validation of the TRPRS scoring system′s high prognostic effect across multiple datasets. Kaplan–Meier survival curves and time‐dependent ROC curves for high‐ and low‐risk groups in TCGA_GBM (a), CGGA_mRNAseq_693 (b), CGGA_mRNAseq_325 (c), GSE13041 (d), and GSE4412 (e).

**Figure:**
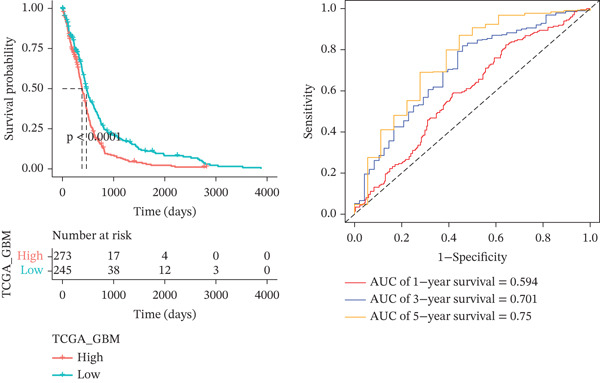
(a)

**Figure:**
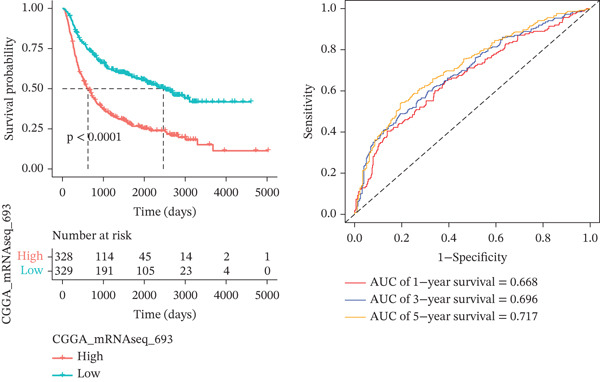
(b)

**Figure:**
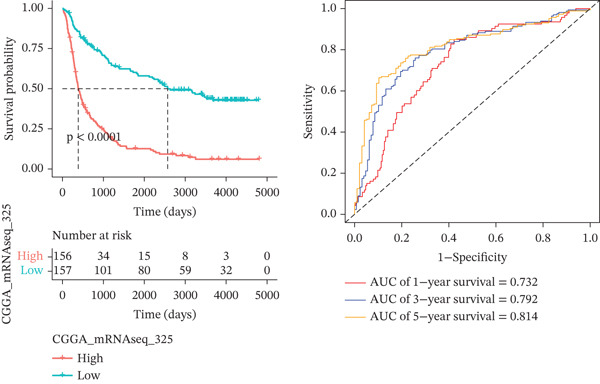
(c)

**Figure:**
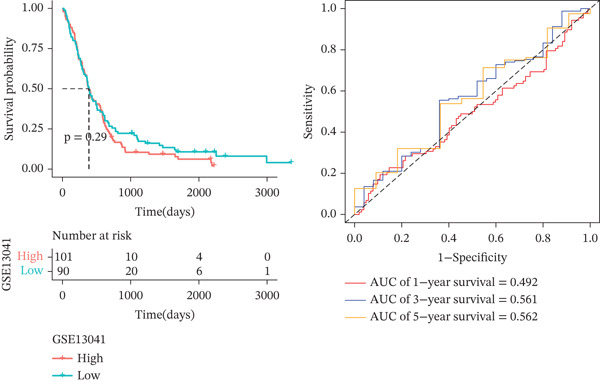
(d)

**Figure:**
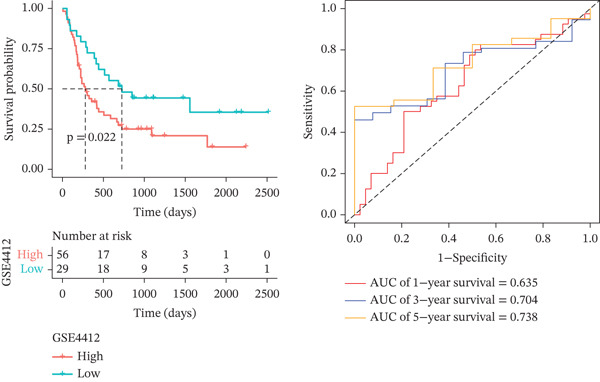
(e)

Figure 5Univariate and multivariate Cox regression analyses of TRPRS prognostic correlation with clinical characteristics. Univariate and multivariate Cox regression analyses of TRPRS with clinical features in TCGA_GBM (a), CGGA_mRNAseq_693 (b), CGGA_mRNAseq_325 (c), GSE13041 (d), and GSE4412 (e).

**Figure:**
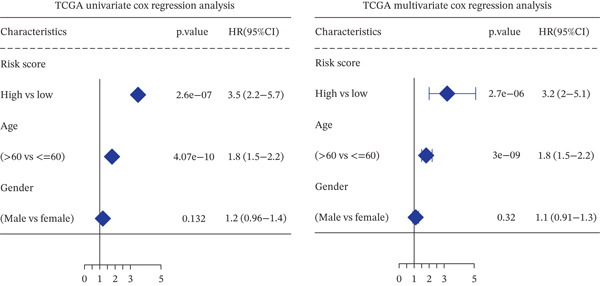
(a)

**Figure:**
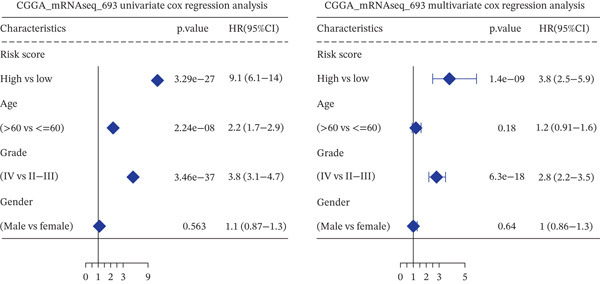
(b)

**Figure:**
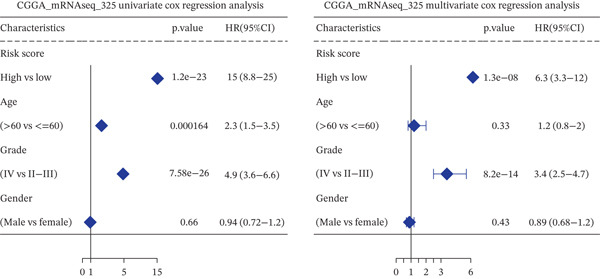
(c)

**Figure:**
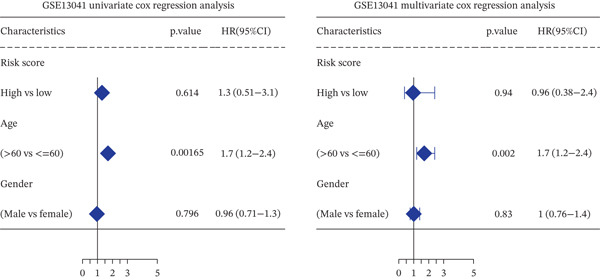
(d)

**Figure:**
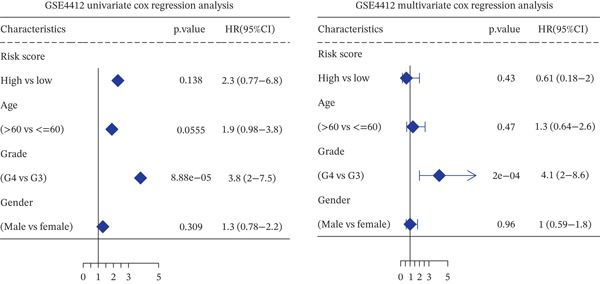
(e)

### 3.5. TRPRS Associates With Clinical Variables, Immune Contexture, and Immune Checkpoints

High‐TRPRS tumors were enriched for MGMT‐unmethylated, IDH1‐wild‐type, and classical subtype features (Figure 6a). Immune infiltration profiling showed lower abundance of CD8^+^ T cells, naïve CD4^+^ T cells, activated dendritic cells, and neutrophils in the high‐risk group (Figure 6b). Expression of immune checkpoints was also linked to TRPRS: positive correlations with CD80/CD86 and negative correlations with CTLA‐4 and PD‐L1 (CD274) (Figure 6c).

Figure 6Association of TRPRS with clinical features, immune infiltration, and immune checkpoints in GBM. (a) Analysis of the significance of differences between TRPRS and 1p_19q codeletion, MGMT methylation status, molecular subtype (original subtype), IDH1 mutation status, age, and gender. (b) Differences in immune infiltration between high TRPRS and low TRPRS groups. (c) Correlation between TRPRS and expression of immune checkpoints ( ^∗^ *p* < 0.05;  ^∗∗^ *p* < 0.01;  ^∗∗∗^ *p* < 0.001; and  ^∗∗∗∗^ *p* < 0.0001; ns *p* > 0.05).

**Figure:**
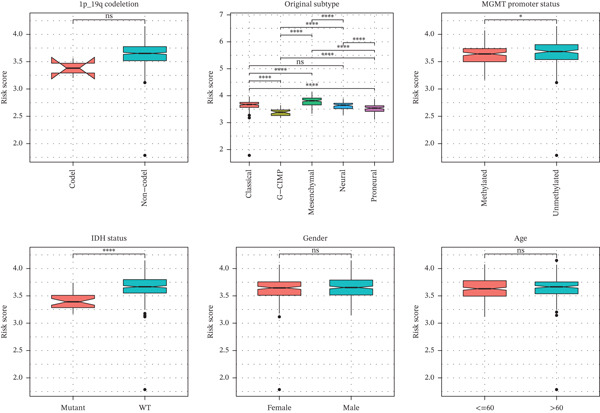
(a)

**Figure:**
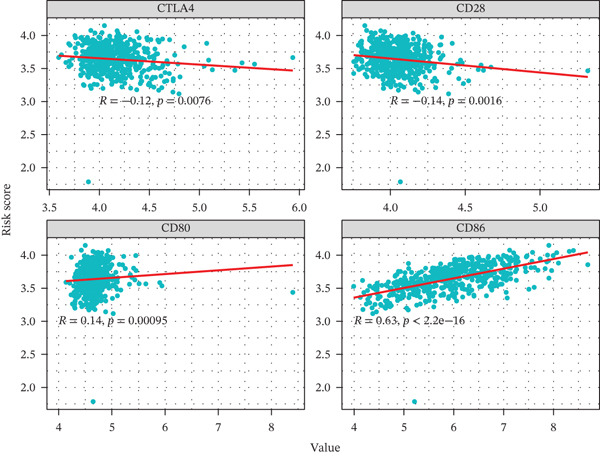
(b)

**Figure:**
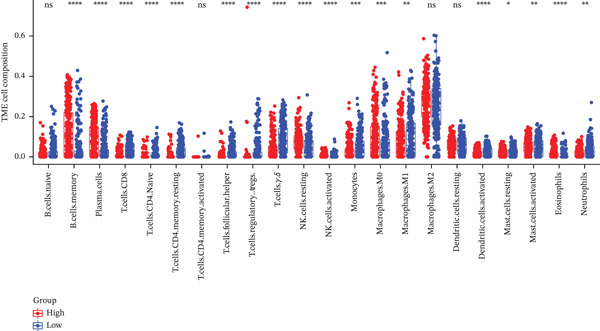
(c)

### 3.6. TRPRS Predicts Chemotherapy and Immunotherapy Response

Using the pRRophetic algorithm, we estimated chemotherapeutic sensitivity for each GBM sample and observed that high‐TRPRS tumors were significantly more resistant to cisplatin, carmustine, gefitinib, buparlisib, afatinib, and several other agents than low‐TRPRS tumors (Figure 7a). To extend these findings to immunotherapy, TIDE scores were calculated for the TCGA‐GBM training set; patients in the high‐TRPRS group exhibited markedly higher TIDE values—indicating a greater likelihood of immune escape—and consequently poorer immunotherapy outcomes (*p* = 5 × 10 − 4, Figure 7b). This predictive capacity was further validated in three independent immunotherapy‐treated cohorts: melanoma (GSE91061, *p* < 0.0001, Figure 7c), renal clear‐cell carcinoma (*p* = 0.0072, Figure 7d,e), and bladder cancer (IMvigor210, *p* = 0.00012, Figures 7f, 7g, and 7h), underscoring the broad utility of TRPRS for forecasting therapeutic response across cancer types.

Figure 7TRPRS as valuable predictors of response to chemotherapy and immunotherapy. (a) Evaluation of chemotherapeutic drug sensitivity in the training set cohort. (b) TIDE analysis of expression profiles of TCGA_GBM subtype samples in the training set. (c) Evaluation of immune therapy prognostic effects using TRPRS in other immune therapy cohorts, including melanoma GSE91061 dataset (c), clear cell renal carcinoma (d, e), and bladder cancer dataset IMvigor210 (f, g, h) ( ^∗^ *p* < 0.05;  ^∗∗^ *p* < 0.01;  ^∗∗∗^ *p* < 0.001; and  ^∗∗∗∗^ *p* < 0.0001; ns *p* > 0.05).

**Figure:**
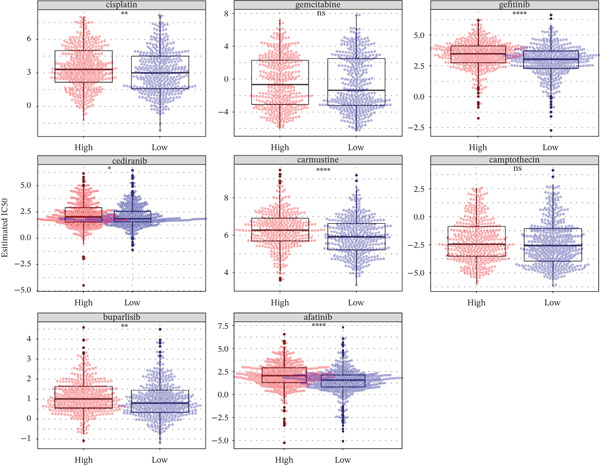
(a)

**Figure:**
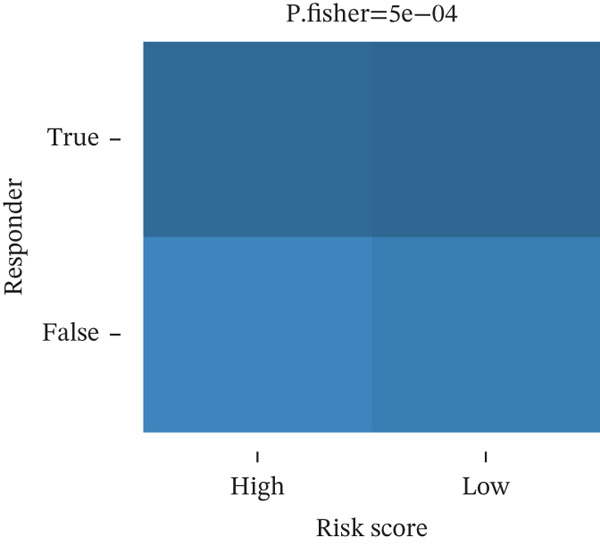
(b)

**Figure:**
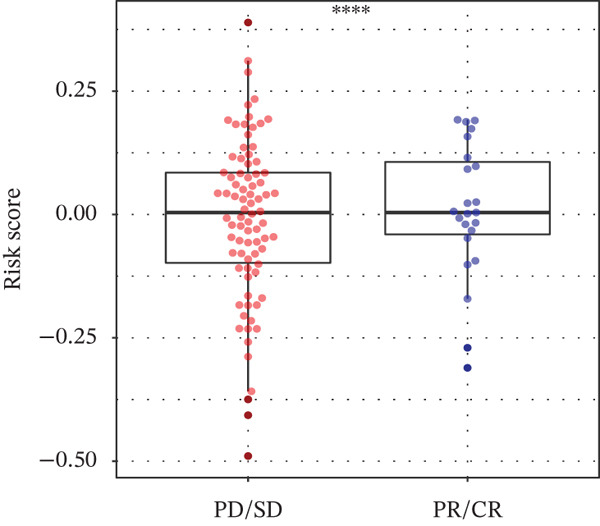
(c)

**Figure:**
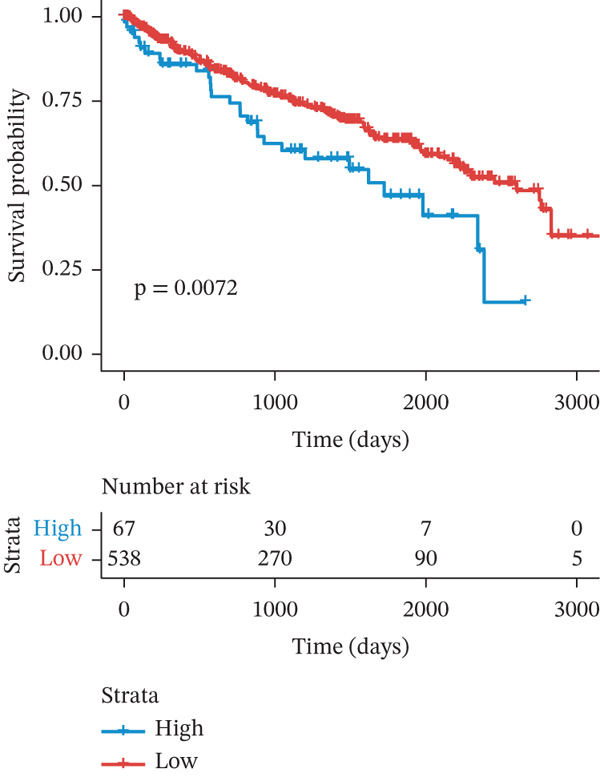
(d)

**Figure:**
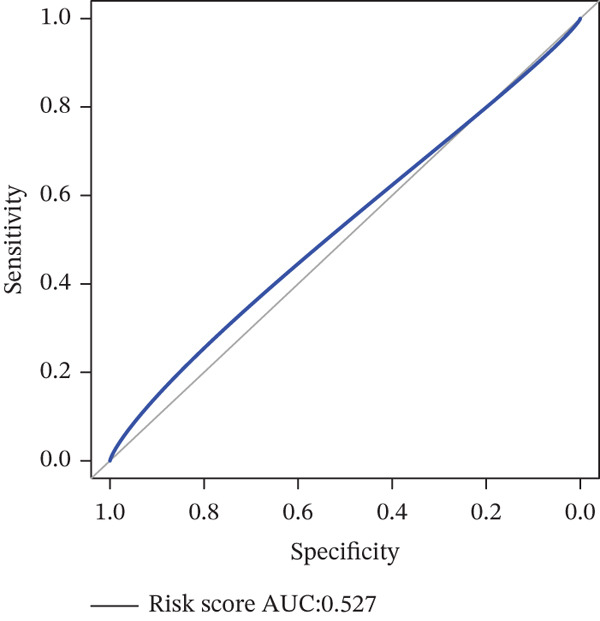
(e)

**Figure:**
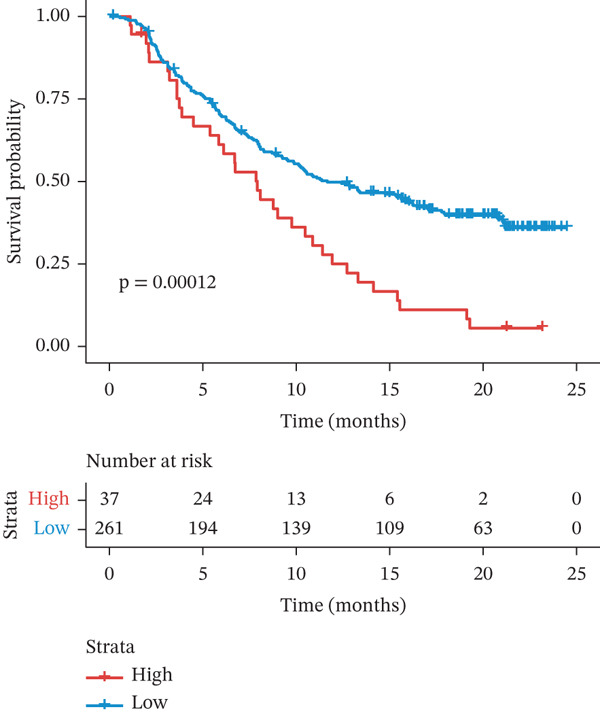
(f)

**Figure:**
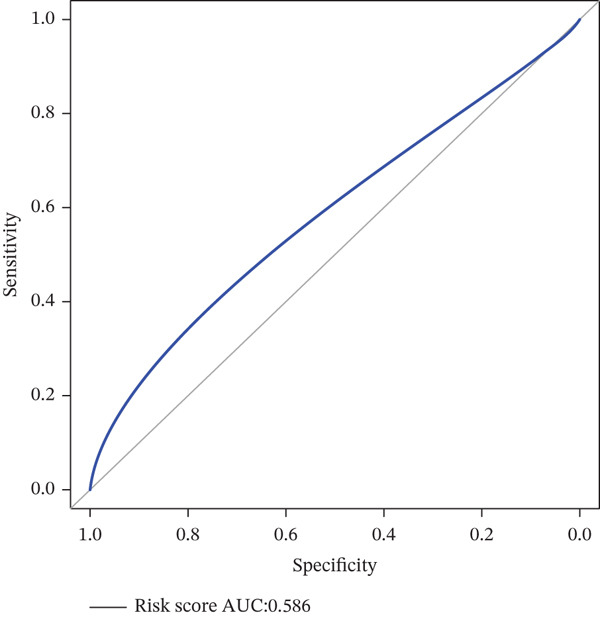
(g)

**Figure:**
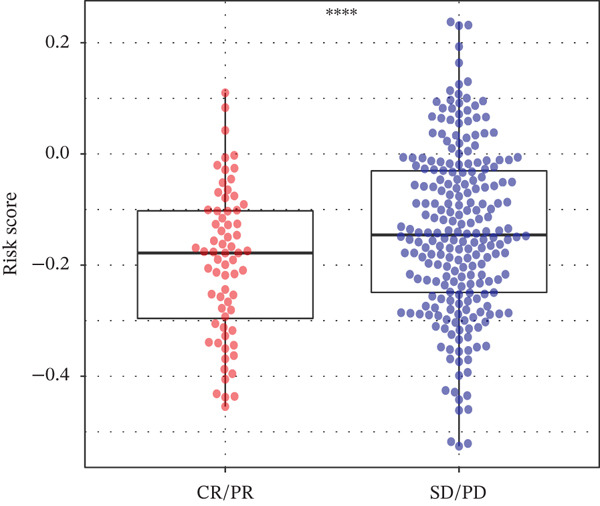
(h)

### 3.7. Distinct Mutation Spectra and Copy Number Landscapes of TRPRS Subgroups

The 20 most frequently mutated genes differed between risk groups: TP53 dominated in low‐TRPRS tumors, whereas PTEN ranked first in high‐TRPRS tumors (Figure 8a,b). GISTIC 2.0 analysis identified 9q21.3 amplification as the most significant focal gain in high‐TRPRS tumors, whereas 1q21.3 deletion was prominent in low‐TRPRS tumors (Figure 8c,d).

Figure 8Identification of TRP‐associated mutant genes and chromosomal locations. (a) The 20 most commonly mutated genes in the low‐risk group. (b) The 20 most commonly mutated genes in the high‐risk group. (c) Detection of significant copy number amplifications and deletions in the low‐risk group. As shown, the vertical coordinate represents the GISTIC score (G‐score), with a G‐score of > 0 indicating amplification and a G‐score of < 0 indicating deletion. The threshold for significance is set at FDR < 0.05. (d) Comparison of copy number amplifications and deletions between the low‐ and high‐risk groups.

**Figure:**
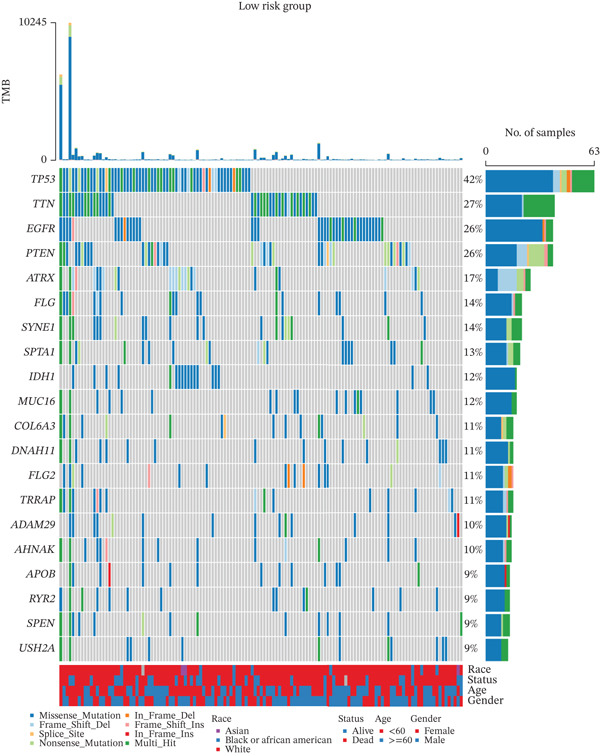
(a)

**Figure:**
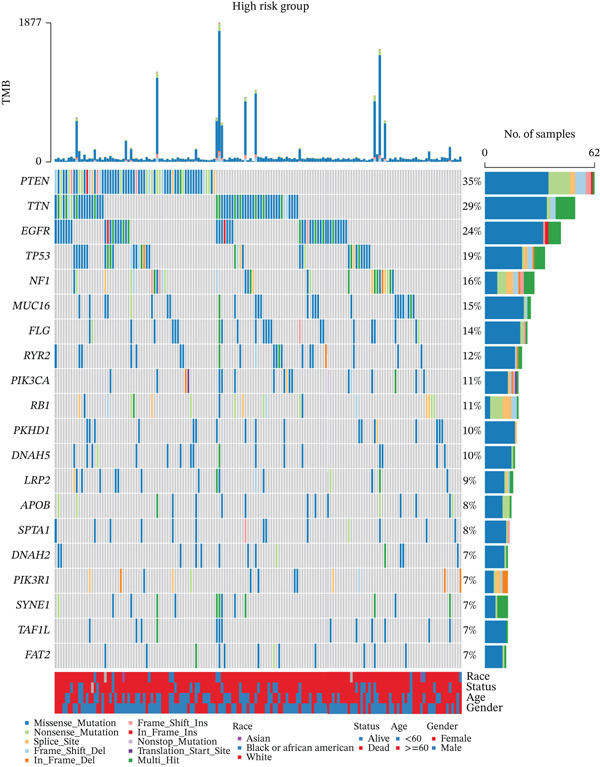
(b)

**Figure:**
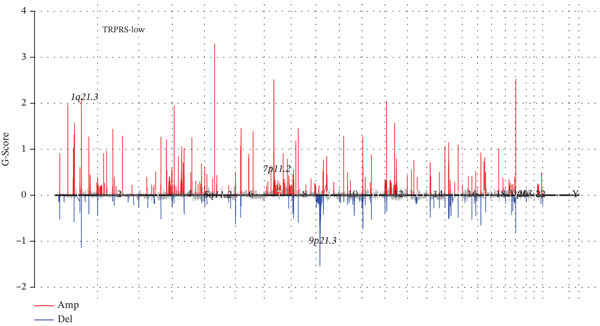
(c)

**Figure:**
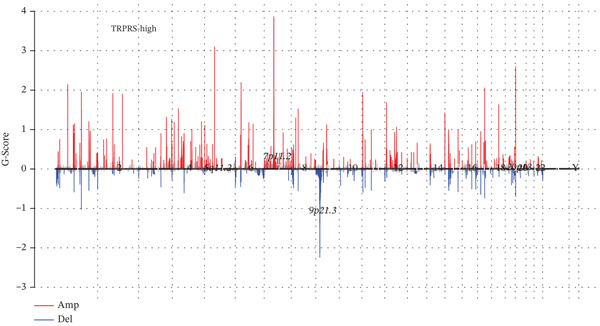
(d)

### 3.8. IFNGR2 Is Overexpressed in GBM and Drives Malignant Phenotypes

We next quantified the transcript levels of the seven core TRP‐signature genes—TNFRSF11B, PKD2, IL1RAP, IFNGR2, HRH1, FCGR2B, and CCL2—in glioma specimens and observed that all were significantly upregulated relative to nontumor brain tissue, with IFNGR2 showing the most pronounced overexpression (Figures 9a, 9b, 9c, 9d, 9e, and 9f). siRNA screening identified si‐IFNGR2_2 as the most efficient duplex (> 70% knockdown; Figures 10a, 10b, 10d, and 10e). Silencing IFNGR2 significantly reduced viability (MTT), proliferation (EdU), and colony formation (Figures 10c, 10f, 10g, 10h, and 10I), whereas lentiviral overexpression in HA cells enhanced proliferation and survival (Figures 10j, 10k, 10l, and 10m).

Figure 9IFNGR2 was upregulated in glioma cell lines. (a–b) PCR detection of transcription levels of TNFRSF11B, PKD2, IL1RAP, IFNGR2, HRH1, FCGR2B, and CCL2 in U87 and U251 cell lines. (c–f) Western‐blot detection of IFNGR2 protein concentration in U87 and U251 cell lines.

**Figure:**
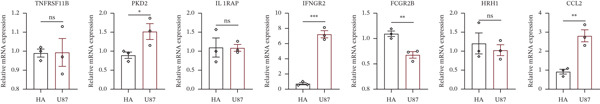
(a)

**Figure:**
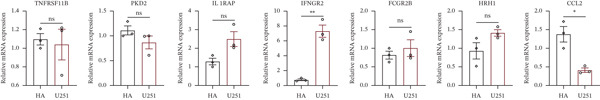
(b)

**Figure:**
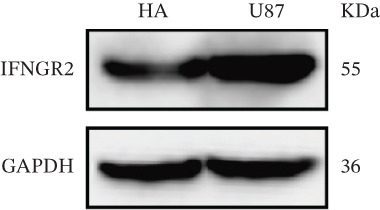
(c)

**Figure:**
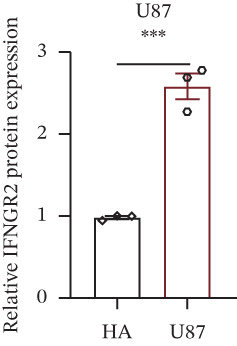
(d)

**Figure:**
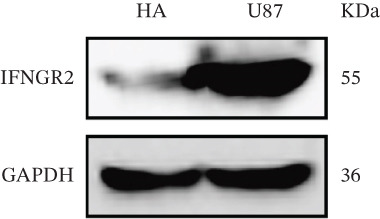
(e)

**Figure:**
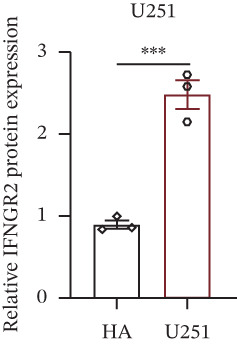
(f)

Figure 10IFNGR2 promotes the malignant phenotype of glioma cell lines. (a–b) The inhibitory efficiency of siRNA in the U87 cell line was detected by PCR and western‐blot. (c) Changes in cell survival in the U87 cell line after inhibition of IFNGR2 expression. (d–e) The inhibitory efficiency of siRNA in the U251 cell line was detected by PCR and western‐blot. (F) Changes in cell survival in the U251 cell line after inhibition of IFNGR2 expression. (g–h) Changes in cell proliferation ability in the U87 and U251 cell lines after inhibition of IFNGR2 expression. (i) Changes in clonogenic ability in the U87 and U251 cell lines after inhibition of IFNGR2 expression. (j) Validation of the efficacy of the IFNGR2 overexpression plasmid in the HA cell line. (k–m) Changes in cell survival and proliferation ability in the HA cell line after overexpression of IFNGR2.

**Figure:**
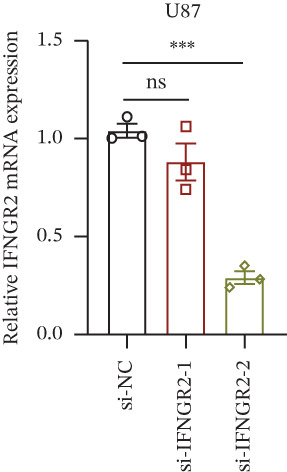
(a)

**Figure:**
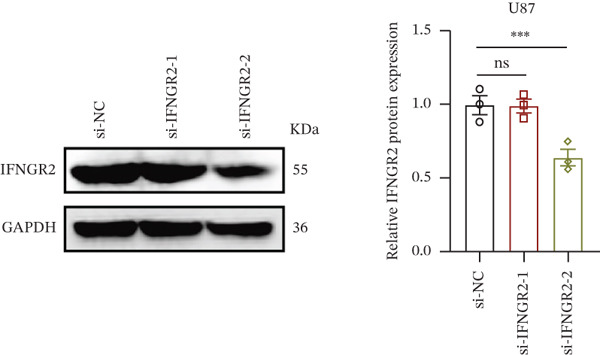
(b)

**Figure:**
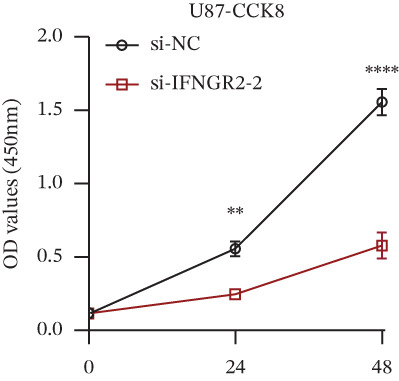
(c)

**Figure:**
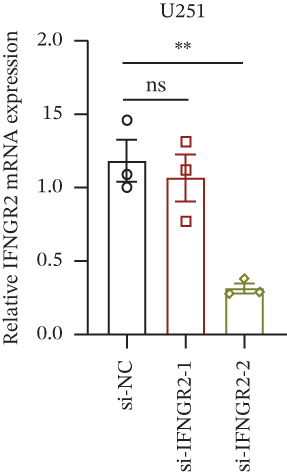
(d)

**Figure:**
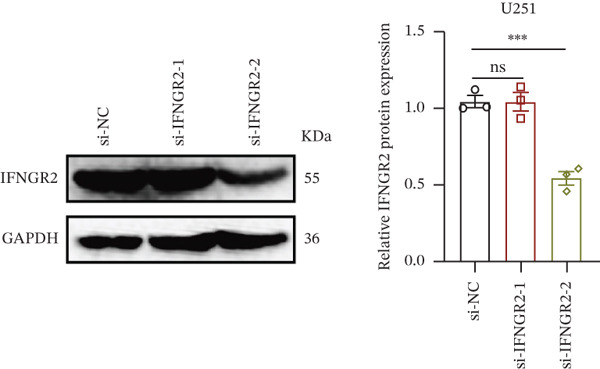
(e)

**Figure:**
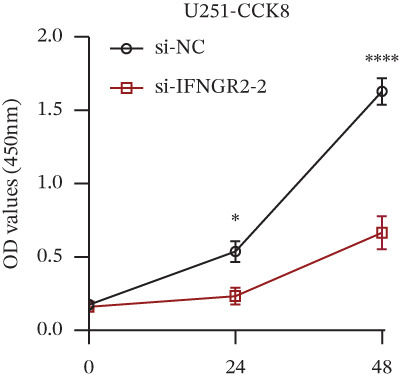
(f)

**Figure:**
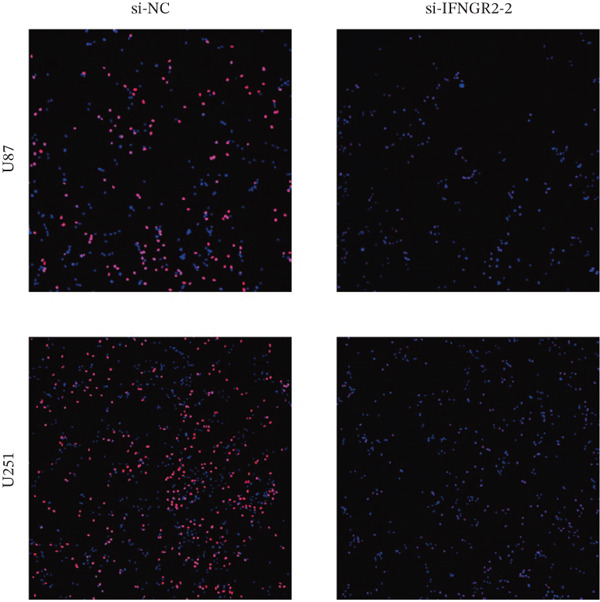
(g)

**Figure:**
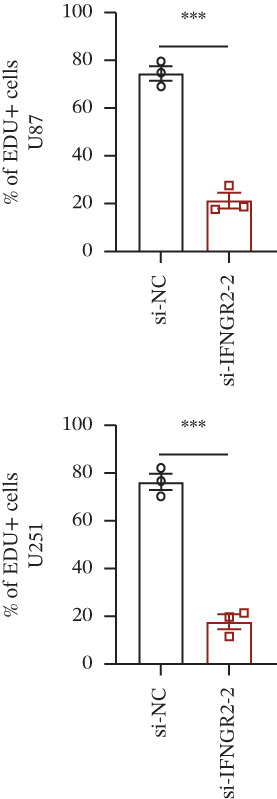
(h)

**Figure:**
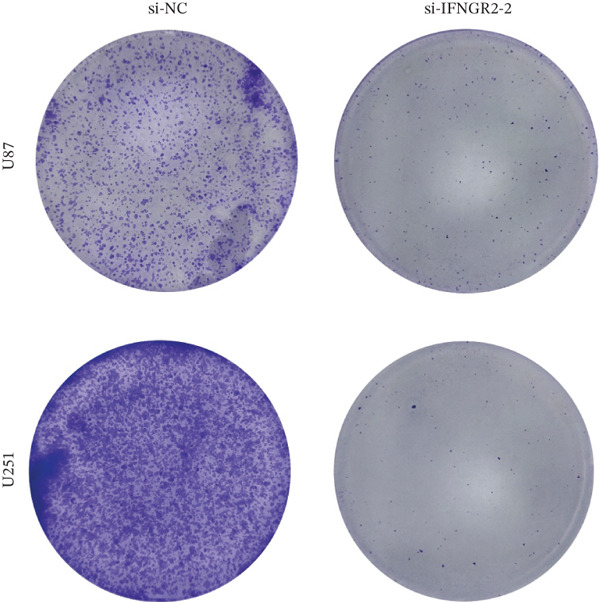
(i)

**Figure:**
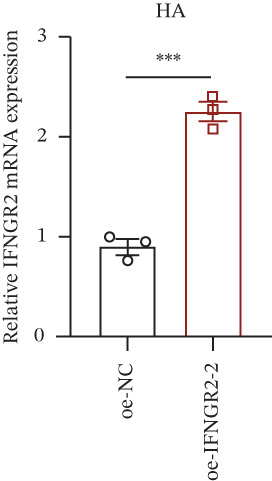
(j)

**Figure:**
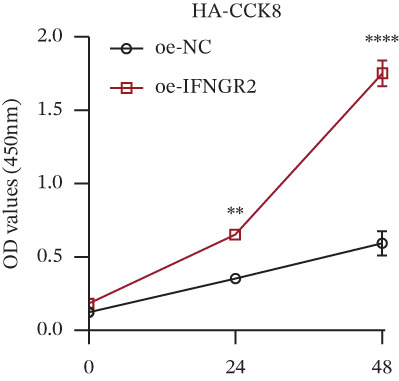
(k)

**Figure:**
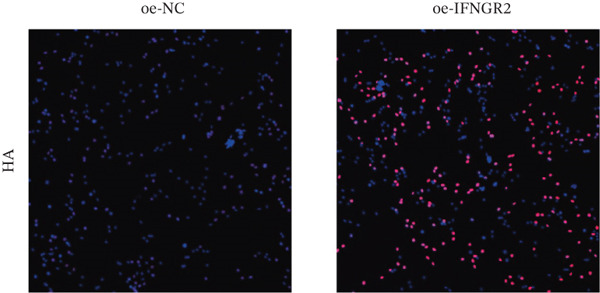
(l)

**Figure:**
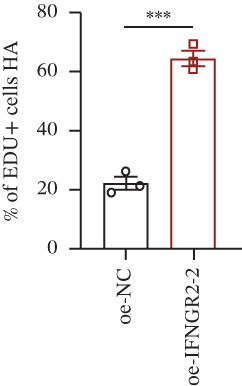
(m)

### 3.9. IFNGR2 Activates NF‐*κ*B Signaling and Instigates a Proinflammatory Tumor Milieu

The knockdown of IFNGR2 in U87/U251 decreased phosphorylation of NF‐*κ*B p50 and p65 subunits, downregulated downstream IL‐6 and TNF‐*α* secretion, and attenuated expression of CXCL1/2/8, thereby impairing the NF‐*κ*B–dependent inflammatory feed‐forward loop that underpins glioma aggressiveness (Figures 11a, 11b, 11c, 11d, 11e, 11f, 11g, 11h, 11i, and 11j).

Figure 11IFNGR2 activates tumor‐related signaling pathways. (a–d) Changes in the phosphorylation levels of p50 and p65 in the U87 cell line after IFNGR2 expression is inhibited. (e) Changes in inflammatory factors in the U87 cell line after IFNGR2 expression is inhibited. (f–i) Changes in the phosphorylation levels of p50 and p65 in the U251 cell line after IFNGR2 expression is inhibited. (j) Changes in inflammatory factors in the U251 cell line after IFNGR2 expression is inhibited.

**Figure:**
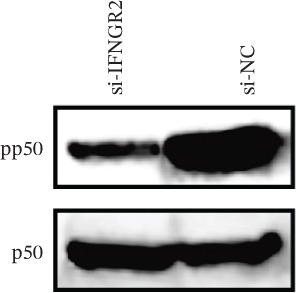
(a)

**Figure:**
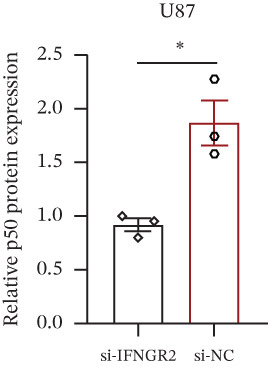
(b)

**Figure:**
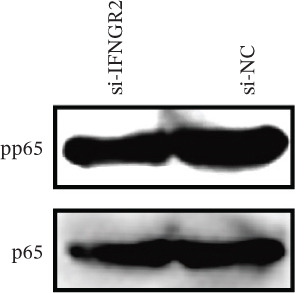
(c)

**Figure:**
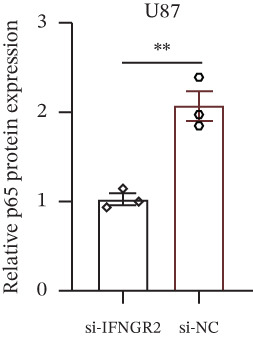
(d)

**Figure:**
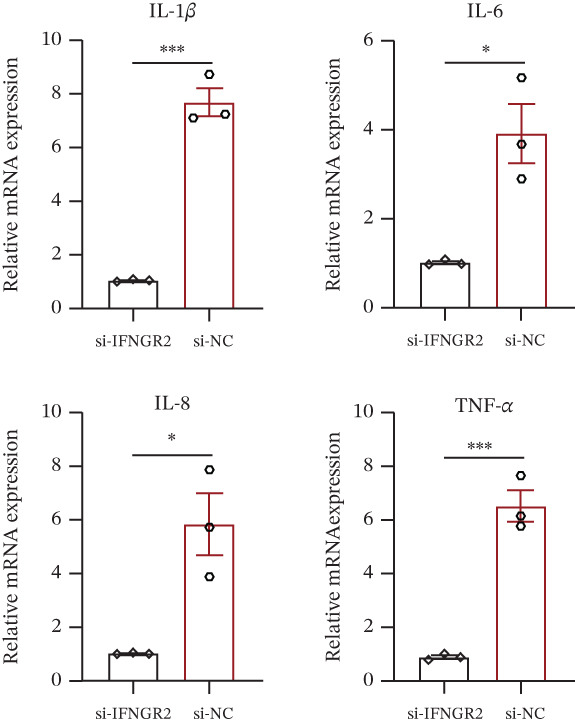
(e)

**Figure:**
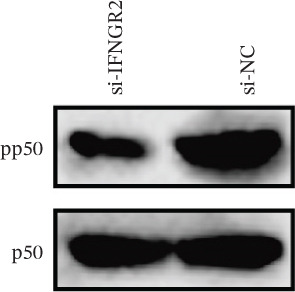
(f)

**Figure:**
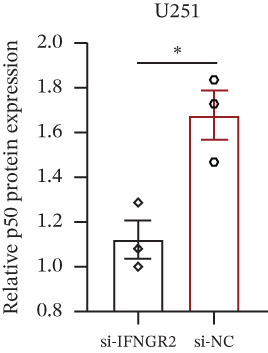
(g)

**Figure:**
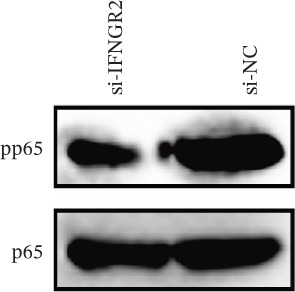
(h)

**Figure:**
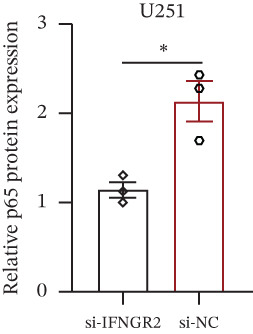
(i)

**Figure:**
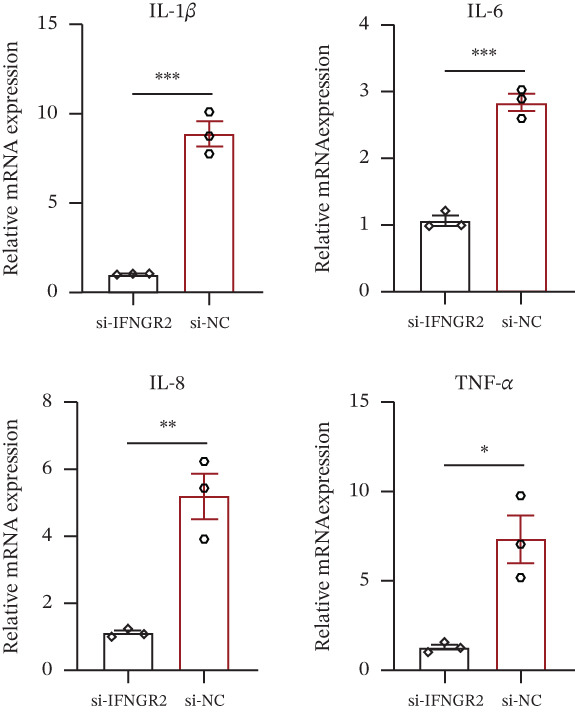
(j)

## 4. Discussion

GBM remains the most lethal primary brain malignancy, with median survival barely exceeding 15 months despite maximal surgical resection, chemoradiation, and adjuvant temozolomide [22, 23]. In this study, we developed a seven‐gene TRPRS derived from an integrative analysis of 522 TRP channel– and pathway‐associated genes. TRPRS robustly stratifies GBM patients into high‐ and low‐risk groups with significantly divergent overall survival and differential responses to immunotherapy across five independent cohorts. Genomically, high‐TRPRS tumors are enriched for PTEN loss and 9q21.3 amplification, whereas low‐TRPRS tumors frequently harbor TP53 mutations and 1q21.3 deletions—providing a molecular framework that underpins their distinct clinical behaviors.

Our multiomics analysis further revealed substantial differences in the genomic architecture between risk groups, including recurrent copy number variations (CNVs), focal amplifications, and deletions. These alterations not only correlate strongly with TRPRS but also offer mechanistic insights into how dysregulated TRP signaling may drive tumor aggressiveness and therapeutic resistance. Notably, high TRPRS is associated with a “cold” immune microenvironment—characterized by reduced cytotoxic T‐cell infiltration—and predicts diminished benefit from immune checkpoint blockade, a finding consistently replicated in melanoma, renal, and bladder cancer immunotherapy cohorts. Future research should focus on expanding the datasets, incorporating additional biomarkers, and exploring the model′s generalizability to enhance its clinical relevance [23–25].

An intriguing finding of our study is the prominent role of IFNGR2—a gene that does not encode a TRP channel but is functionally linked to TRP‐mediated signaling—in shaping GBM aggressiveness. Although IFNGR2 encodes the *β*‐subunit of the interferon‐*γ* (IFN‐*γ*) receptor, its selection in the TRPRS highlights a previously underappreciated crosstalk between Ca^2+^‐dependent TRP signaling and cytokine‐driven inflammatory pathways in glioma. Accumulating evidence indicates that several TRP channels, including TRPC1, TRPV2, and TRPM7, are aberrantly expressed in GBM and regulate intracellular Ca^2+^ dynamics that directly modulate NF‐*κ*B activity—a master transcription factor governing inflammation, survival, and therapy resistance [12, 26]. Notably, NF‐*κ*B can transcriptionally upregulate components of the IFN‐*γ* signaling cascade, including IFNGR1 and IFNGR2, thereby amplifying cellular responsiveness to IFN‐*γ* in the tumor microenvironment [27, 28]. Our functional experiments demonstrate that IFNGR2 knockdown significantly impairs glioma cell proliferation and suppresses NF‐*κ*B phosphorylation, suggesting a reciprocal positive feedback loop: TRP‐mediated Ca^2+^ influx activates NF‐*κ*B, which in turn enhances IFN‐*γ* receptor expression and heightens cellular sensitivity to microenvironmental IFN‐*γ*, subsequently reinforcing NF‐*κ*B signaling.

Nevertheless, TRPRS represents an immediately deployable, biologically grounded tool for personalized risk assessment and treatment selection in GBM. By implicating TRP signaling—a pathway not traditionally linked to gliomagenesis—as a central regulator of prognosis, immune evasion, and drug response, our work uncovers a novel therapeutic vulnerability. Future studies should focus on prospective validation, integration of additional biomarkers (e.g., epigenetic or proteomic signatures), and expansion to diverse, multi‐institutional cohorts to enhance generalizability.

Despite its strengths, our model has limitations. First, it was developed using retrospective data and has not yet undergone prospective clinical validation. Second, potential biases related to ethnicity, treatment heterogeneity, or unmeasured host factors (e.g., systemic immune status) were not fully accounted for. Third, although our genomic profiling is comprehensive, the extreme intratumoral heterogeneity of GBM may obscure rarer but biologically relevant alterations. Finally, the current model does not dynamically incorporate evolving features such as longitudinal changes in the tumor microenvironment or prior therapeutic exposures. In the future, based on our previous work, we will try to use a larger human genome database for validation [29]. Targeting TRP‐related genes for the treatment of glioblastoma is also being explored in our future research [30].

In summary, our findings advance the paradigm of precision neuro‐oncology by linking TRP‐related molecular signatures to clinically actionable outcomes. TRPRS not only improves prognostic accuracy but also informs immunotherapy and targeted therapy decisions, thereby supporting the development of individualized treatment strategies. While ongoing refinement is warranted, this framework brings us closer to the ultimate goal of optimizing therapeutic efficacy and extending survival of GBM patients.

## Author Contributions

Writing–original draft: R.S., L.Z., Z.L., Z.W., S.F., and J.Z. Conceptualization: R.S., L.Z., S.F., and J.Z. Writing–review & editing: S.F. and J.Z. Methodology: R.S., L.Z., Z.L., Z.W., S.F., and J.Z. Resources: J.Z. Funding acquisition: R.S. and S.F. Data curation: L.Z., Z.L., and Z.W. Validation: Z.L. and Z.W. Supervision: S.F. and J.Z. Formal analysis: Z.L. Experiment: Z.L. Project administration: R.S., S.F., and J.Z. Visualization: Z.L. and Z.W. R.S. and L.Z. have contributed equally to this work and share first authorship.

## Funding

This study was supported by Wuxi Municipal Health Commission′s Research Project Plan (Q202120).

## Disclosure

All authors have read and agreed to the published version of the manuscript.

## Conflicts of Interest

The authors declare no conflicts of interest.

## Supporting information


**Supporting Information** Additional supporting information can be found online in the Supporting Information section. Primer pairs and antibody information were listed in the Supporting Information.

## Data Availability

All data used in this study are available in the public repository. The data that support the findings of this study are openly available in TCGA at https://portal.gdc.cancer.gov/ and GEO at http://www.ncbi.nlm.nih.gov/geo/.
