# Supplementary material for: Prognostic Stratification and Subtyping of Glioblastoma Using Transient Receptor Potential Channels
Source: Hum Mutat. 2026 Apr 19;2026:7039302. doi: 10.1155/humu/7039302 (PMC13092933; doi:10.1155/humu/7039302)
Supplement: Supplementary file 1 — Supporting Information Additional supporting information can be found online in the Supporting Information section. Primer pairs and antibody information were listed in the Supporting Information. [file HUMU-2026-7039302-s001.docx]

Primer pairs

| Gene name | Forward primer (5’-3’) | Reverse primer |
| --- | --- | --- |
| TNFRSF11B | GGTCTCCTGCTAACTCAGAAAGG | CAGCAAACCTGAAGAATGCCTCC |
| PKD2 | AAATGTCTGGCTGGACCGAGGA | GGAATCACACCACCTGTTGCTG |
| IL1RAP | CTGAGGATCTCAAGCGCAGCTA | AGCAGGACTGTGGCTCCAAAAC |
| IFNGR2 | CTCCATTCTGCCTGGGTGACAA | CGTGGAGGTATCAGCGATGTCA |
| HRH1 | GGATGCCAAGAAACCAGGGAAG | CTCTTGGCTGAAGACAACTGGG |
| FCGR2B | CTACTGTGGACAGCCGTGCTAA | TCACCGTGTCTTCCTTGAGCAC |
| CCL2 | AGAATCACCAGCAGCAAGTGTCC | TCCTGAACCCACTTCTGCTTGG |
| IL-1β | CCACAGACCTTCCAGGAGAATG | GTGCAGTTCAGTGATCGTACAGG |
| IL-6 | AGACAGCCACTCACCTCTTCAG | TTCTGCCAGTGCCTCTTTGCTG |
| IL-8 | GAGAGTGATTGAGAGTGGACCAC | CACAACCCTCTGCACCCAGTTT |
| TNF-α | CTCTTCTGCCTGCTGCACTTTG | ATGGGCTACAGGCTTGTCACTC |
| GAPDH | GGAGTCCACTGGTGTCTTCA | GGGAACTGAGCAATTGGTGG |

Antibody information

| Proteins | Host | KDa | Catalog | Antibody dilution |
| --- | --- | --- | --- | --- |
| IFNGR2 | Rabbit | 55 | 10266-1-AP | 1:1000 |
| p50 | Rabbit | 50 | T55040S | 1:5000 |
| p50 (Ser337) | Rabbit | 50 | TA3219S | 1:1000 |
| p65 | Rabbit | 65 | T55034S | 1:5000 |
| p65 (Ser536) | Rabbit | 65 | TP56372S | 1:1000 |
| GAPDH | Rabbit | 36 | 81640-5-RR | 1:10000 |
